# A model for isoform-level differential expression analysis using RNA-seq data without pre-specifying isoform structure

**DOI:** 10.1371/journal.pone.0266162

**Published:** 2022-05-16

**Authors:** Yang Liu, Junying Wang, Song Wu, Jie Yang

**Affiliations:** 1 Department of Applied Mathematics and Statistics, Stony Brook University, Stony Brook, NY, United States of America; 2 Department of Family, Population, Preventive Medicine, School of Medicine, Stony Brook University, Stony Brook, NY, United States of America; Icahn School of Medicine at Mount Sinai, UNITED STATES

## Abstract

**Motivation:**

Next generation sequencing (NGS) technology has been widely used in biomedical research, particularly on those genomics-related studies. One of NGS applications is the high-throughput mRNA sequencing (RNA-seq), which is usually applied to evaluate gene expression level (i.e. copies of isoforms), to identify differentially expressed genes, and to discover potential alternative splicing events. Popular tools for differential expression (DE) analysis using RNA-seq data include edgeR and DESeq. These methods tend to identify DE genes at the gene-level, which only allows them to compare the total size of isoforms, that is, sum of an isoform’s copy number times its length over all isoforms. Naturally, these methods may fail to detect DE genes when the total size of isoforms remains similar but isoform-wise expression levels change dramatically. Other tools can perform isoform-level DE analysis only if isoform structures are known but would still fail for many non-model species whose isoform information are missing. To overcome these disadvantages, we developed an isoform-free (without need to pre-specify isoform structures) splicing-graph based negative binomial (SGNB) model for differential expression analysis at isoform level. Our model detects not only the change in the total size of isoforms but also the change in the isoform-wise expression level and hence is more powerful.

**Results:**

We performed extensive simulations to compare our method with edgeR and DESeq. Under various scenarios, our method consistently achieved a higher detection power, while controlling pre-specified type I error. We also applied our method to a real data set to illustrate its applicability in practice.

## 1. Introduction

Next-generation sequencing technology (NGS) has been widely used in genomic studies [1]. One of its applications, the whole transcriptome sequencing (RNA-seq), makes it available to evaluate gene expression level and discover potential alternative splicing events by generating high-throughput sequencing data [2–4]. RNA-seq has shown the ability to create highly accurate and replicable genetic data compared with traditional microarrays [5, 6]. There are two types of RNA-seq data, single-end and paired-end. The single-end reads come from sequencing one end of the RNA-seq fragments while the paired-end reads come from sequencing both ends of the RNA-seq fragments.

Nowadays, differential expression (DE) analysis using RNA-seq data is one of the most popular research areas and various statistical methodologies have been proposed for detecting differentially expressed genes. Currently edgeR [7] and DESeq [8] are two popular R packages for detecting DE genes at the gene-level. Because of ignoring the isoform structures, these two methods are only sensitive to the change in the total size of isoforms, that is, sum of an isoform’s copy number times its length over all isoforms. Since different isoforms are translated to different polypeptides, changes in the expression level of isoforms may cause the functional shift of a gene. Thus, a gene should be considered as differentially expressed if any of its isoforms is differentially expressed. It may happen that the total size of isoforms from one specific gene under different conditions are very similar but the isoform-wise copies are totally different. In such situation, a gene-level analysis would miss this type of isoform-wise DE difference.

Some other methods have been proposed to provide the isoform-level analysis by pre-specifying isoform structures [9, 10]. However, these methods may suffer when isoform structures are limited, and may not even be applicable for many species, like non-model animals and plants, whose isoform information are greatly missing. In addition, since reads from RNA-seq data are usually short (~100bp), it is very hard to rebuild the complete isoform structure even when exon structures are known.

In this paper, we propose an isoform-free model for identifying DE genes at the isoform-level using RNA-seq reads without pre-specifying any isoform structures. We show that our model achieves a higher detection power than edgeR and DESeq through extensive simulations while controlling pre-specified type-I error.

## 2. Methods

Since NGS reads are independently sequenced during the sequencing procedure, hypothesis testing for DE analyses can then be performed separately for each gene. For simplicity, we describe our model by focusing on modeling RNA-seq data sequenced from one specific gene, e.g. gene g. Also, we focus on using single-end RNA-seq data, as paired-end reads can be analyzed by treating the pair as two single-end reads.

### 2.1 Model

Firstly, we introduce a mechanism to summarize each RNA-seq read to a read type using the definition below, which is the key to perform an isoform-level analysis without any pre-specified isoform structure information.

*Definition 1*. Assume exons are coded as sequential integers starting from 1 (e.g. 1, 2, 3 …). For a specific RNA-seq read, its read type is defined as a string of ordered exon IDs, to which the read is mapped. For example, read type "1" means the read is mapped to exon 1; read type "1–2" means one part of the read is mapped to exon 1 and the other part is mapped to exon 2.

**Fig 1:**
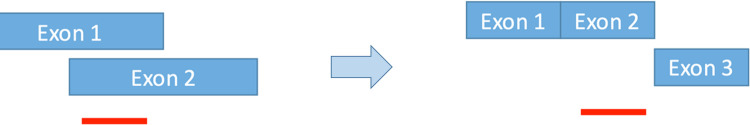
Overlapping exons from a gene annotation file (left) are converted to non-overlapping exons (right). The read type of a RNA-seq read (the red bar) may be ambiguous in the original annotation file but is unique in our converted file.

Read type of each read can be found by using the exons’ information in a gene annotation file. Since there may be overlaps between exon regions in the annotation file, we need to process the file to re-group exons into un-overlapped exons, so that no ambiguous read type can occur when reads are tagged with read types. Fig 1 shows an example that when exon 1 and exon 2 have overlapping region, a read (the red bar) that is mapped to the overlapping region would not have a clear definition of its read type. However, after processing them into non-overlapping exons, the read can be defined as read type "2" without ambiguity.

Secondly, we introduce the notations. Let gene *g*, be any gene from a set of distinct genes {1,…, *G*} and it has *I*_*g*_ distinct isoforms. For each sample *j*, *j* = 1,…, J, let *X*_*gihj*_ be the number of type *h* read sequenced from isoform *i* of gene *g* in sample *j*, *h* = 1…. *H*_*g*_. Let *N*_*j*_ be the total number of mapped reads from sample *j* and let *k*_*gi*_ be the number of copies of isoform *i* of gene *g*. *l*_*gih*_ represents the number of nucleotides which can be the starting positions of type *h* read on isoform *i* of gene *g*, and $l_{gi.}=\sum_{h=1}^{Hg}l_{gih}$. Note that since all isoforms share the same underlying exon structure, for a specific read type *h*, the number of nucleotides that can be the starting positions of this read type would be the same for each isoform that can generate this read type. Therefore, *l*_*gih*_ can only take two values, either 0 if isoform *i* can’t generate read type *h*, or *l*_*g*.*h*_ if isoform *i* can generate read type *h*.

Following the assumption that the sequencing process is a simple random sampling [9], we assume

$$X_{gihj}\sim Poisson\left(N_{j}\frac{k_{gi}l_{gih}}{\sum_{g=1}^{G}\sum_{i=1}^{I_{g}}k_{gi}l_{gi.}}\right).$$

Let $S=\sum_{g=1}^{G}\sum_{i=1}^{I_{g}}k_{gi}l_{gi.}$, and *p*_*gih*_ = *l*_*gih*_/*l*_*gi*._, we have

$$X_{gihj}\sim Poisson\left(N_{j}\frac{p_{gih}k_{gi}l_{gi.}}{S}\right).$$

*S* represents the overall transcriptome size and *p*_*gih*_ is the probability of getting a type *h* read given that the read is sequenced from isoform *i*. However, *X*_*gihj*_ is not observable in practice, instead, only *X*_*ghj*_ is observable with a different Poisson distribution under the simple random sampling assumption:

$$X_{ghj}=\overset{I_{g}}{\underset{i=1}{\sum }}X_{gihj}\sim Poisson\left(N_{j}\frac{\sum_{i=1}^{I_{g}}p_{gih}k_{gi}l_{gi.}}{S}\right).$$

In order to reduce the bias caused by different transcriptome sizes and to adapt the variation due to biological replication, we propose to adjust the transcriptome size with trimmed mean of M values (TMM) normalization [11]and extend the distribution assumption of *X*_*ghj*_ from Poisson distribution to Negative Binomial distribution [12, 13]. That is, the copy number *k*_*gi*_ is not a constant but a random variable *K*_*gij*_, whose expectation is *k*_*gi*_.

$$X_{ghj}|\{K_{gij,\;i=1,\ldots ,\;Ig}\}\sim Poisson\left(N_{j}\frac{\sum_{i}p_{gih}K_{gij}l_{gi.}}{S_{j}}\right)$$ (1)

where $S_{j}=\sum_{g=1}^{G}\sum_{i=1}^{Ig}k_{gij}l_{gi.}$, overall transcriptome size for sample *j*.

Following general notations in TMM normalization, (1) can be re-expressed as $X_{ghj}|\Theta_{ghj}\sim Poisson\left(N_{j}^{*}\Theta_{ghj}\right)$, where $N_{j}^{*}=N_{j}\times S_{r}/S_{j},\;S_{r}$, is the transcriptome size of a reference (or base-line) sample *r*, and Θ_*ghj*_ = Σ_*i*_ *p*_*gih*_ *K*_*gij*_*l*_*gi*_ /*S*_*r*_. We assume that for any sample *j*, Θ_*ghj*_ follows a Gamma distribution with the mean equaling Σ_*i*_ *p*_*gih*_ *k*_*gi*_ *l*_*gi*._ /*S*_*r*_ and the shape parameter equaling 1/*φ*_*gh*_. By integrating out Θ and assuming *S*_*j*_ is a constant number, we can get the marginal distribution of *X*_*ghj*_, that is

$$X_{ghj}\sim NB\left(N_{j}^{*}\theta_{gh},\varphi_{gh}\right),$$ (2)

where *θ*_*gh*_ = *E*[Θ_*ghj*_] = Σ_*i*_ *p*_*gih*_ *k*_*gi*._ *l*_*gi*_ /*S*_*r*_. *θ*_*gh*_ can be treated as a relative expression level of type *h* read without adjusting for its length *l*_*gi*._.*φ*_*gh*_ is generally assumed to be the same across different experiment conditions.

Suppose we have two experimental conditions, i.e. 0 and 1, shown as the superscript. The analysis objective is to test if each isoform of a given gene *g* has different expression level under two conditions with the null hypothesis stated below

$$H_{0}:k_{gi}^{0}=k_{gi}^{1},\;i=1,\;2,\;\ldots ,\;I_{g},$$ (3)

where {1,…, *I*_*g*_} is a set of all distinct isoforms of gene *g* and $k_{gi}^{0},\;k_{gi}^{1}$ are the expected expression levels of isoform *i* of gene *g* under condition 0 and 1, respectively. This null hypothesis by using model (2) can be re-written as

$$H_{0}:\theta_{gh}^{0}=\theta_{gh}^{1},\;h=1,\;2,\;\ldots ,\;H_{g},$$ (4)

where {1,…*H*_*g*_} is the set of all distinct read types that can be generated by gene *g*’*s* isoforms. From the negative binomial model (2), we can describe the relationship between the isoform expression level *k*_*gi*_ and the read type expression level *θ*_*gh*_ in a matrix form, that is

$$\vec{\theta_{g}}=\left[\begin{array}{c} \theta_{g1}\\ \vdots \\ \theta_{gH_{g}} \end{array}\right]=\left[\begin{array}{ccc} p_{g11} & \cdots & p_{gI_{g}1}\\ \vdots & \ddots & \vdots \\ p_{g1H_{g}} & \cdots & p_{gI_{g}H_{g}} \end{array}\right]\left[\begin{array}{c} \frac{k_{g1}l_{g1.}}{S_{r}}\\ \vdots \\ \frac{k_{gI_{g}}l_{gI_{g}.}}{S_{r}} \end{array}\right]=P_{g}\vec{k_{g}}$$

For two experiment conditions (e = 0 or 1), we have

$$\vec{\theta_{g}^{e}}=\left[\begin{array}{c} \theta_{g1}^{e}\\ \vdots \\ \theta_{gH_{g}}^{e} \end{array}\right]\;\text{and}\;\vec{k_{g}^{e}}=\left[\begin{array}{c} \frac{k_{g1}^{e}l_{g1.}}{S_{r}}\\ \vdots \\ \frac{k_{gI_{g}}^{e}l_{gI_{g}.}}{S_{r}} \end{array}\right]$$

If the rank of matrix *P*_*g*_ equals the number of its columns, i.e. *P*_*g*_ is a full column rank matrix, then $\vec{k_{g}^{0}}=\vec{k_{g}^{1}}$ if and only if $\vec{\theta_{g}^{0}}=\vec{\theta_{g}^{1}}$.

### 2.2 Parameter estimation and statistical inference

With each observed *X*_*ghj*_ and its marginal distribution (2), we can use the quantile-adjusted conditional maximum likelihood (CML) to estimate the parameter *φ*_*gh*_ [13] and EM algorithm to estimate the parameter *θ*_*gh*_ for a given *φ*_*gh*_. Specifically, if all samples have the same total number of sequenced reads, i.e. $N_{j}^{*}=N^{*}$ for all *j*, we can eliminate the *θ*_*gh*_ by conditioning on its sufficient statistic, $Z_{gh}=\sum_{j=1}^{J}X_{ghj}\sim NB\left(JN^{*}\theta_{gh},J^{-1}\varphi_{gh}\right)$, and get the conditional likelihood function of *φ*_*gh*_,

$$l_{X_{ghj}|Z_{gh}=z}=\left[\overset{J}{\underset{j=1}{\sum }}log\Gamma \left(x_{ghj}+\varphi_{gh}^{-1}\right)\right]+log\Gamma \left(J\varphi_{gh}^{-1}\right)-log\Gamma \left(z+J\varphi_{gh}^{-1}\right)-Jlog\Gamma \left(\varphi_{gh}^{-1}\right)$$

So we could iteratively generate pseudo data $\left(X_{ghj}^{pseudo}\right)$ which have the same $N_{j}^{*}$ and update $\hat{\varphi_{gh}}$ until $\hat{\varphi_{gh}}$ converges. During this procedure, *θ*_*gh*_ needs to be estimated in order to create pseudo data. EM algorithm will be used to update *θ*_*gh*_ for a fixed $\hat{\varphi_{gh}}$. Detailed steps for parameter estimation can be found in A1 in S1 File.

An exact test based on the pseudo data mentioned above can be used for hypothesis testing [13] Now we have $X_{ghj}^{e,pseudo}\sim NB\left(N^{*}\theta_{gh}^{e},{\hat{\varphi }}_{gh}\right)$ and $Z_{gh}^{e}=\overset{J_{e}}{\underset{j=1}{\sum }}X_{ghj}^{e,\;pseudo}\sim NB\left(J_{e}N^{*}\theta_{gh}^{e},J_{e}^{-1}{\hat{\varphi }}_{gh}\right)$, where {1,…, *J*_*e*_} is a set of all samples under condition *e*.

Under *H*_0_,

$$Z_{gh}=Z_{gh}^{0}+Z_{gh}^{1}\sim NB\left(\left(J_{0}+J_{1}\right)N^{*}\theta_{gh},{\left(J_{0}+J_{1}\right)}^{-1}{\hat{\varphi }}_{gh}\right)$$

P-value from this exact test is based on the conditional distribution of $Z_{gh}^{0}|Z_{gh}$ for a single read type *h*. Details can be found in A2 in S1 File.

P-value for gene *g* at the gene level can be obtained from using Bonferroni method on all p-values of its read types. That is, a gene *g* will be identified as a DE gene if any of its read type has a p-value smaller than pre-specified type I error divided by the number of read types.

### 2.3 Model simplification

In lieu of a large number of hypothesis tests for each gene (50–100 tests on average) due to the abundance of different read types, we introduce a splicing graph-based model, which aims to reduce the computational complexity while maintaining type-I error control and power.

Recall that, as long as matrix *P*_*g*_ is a full column rank matrix, null hypothesis (3) and (4) are equivalent. Elements in matrix *P*_*g*_ are

$$p_{gih}=\frac{l_{gih}}{l_{gi.}}=\left\{\begin{array}{c} \frac{l_{g.h}}{l_{gi.}},\;if\;isoform\;i\;can\;generate\;h\\ 0,\;if\;isoform\;i\;can^{\prime }t\;generate\;h \end{array}\right.$$

Therefore, we want to reduce the number of read types, but keep the column rank of *P*_*g*_ as full. We know that, if the corresponding rows of two read types in *P*_*g*_ are proportional to each other, then after summing them together, both row rank and column rank of *P*_*g*_ do not change. Summing two rows together in *P*_*g*_ is equivalent to combine two read types together to make a new type of read, and this leads to a reduction in the number of read types from two to one.

*Definition 2*. *Given two read types h and h’*, *if the set of isoforms that can generate h is the same as the set of isoforms that can generate h’*, *we call these two read types ‘always showing together’*.

For any two read types *h* and *h*’ that are always showing together, their corresponding rows in *P*_*g*_ are listed below

$$\left[\begin{array}{cc} \begin{array}{cc} \frac{l_{g.h}}{l_{g1.}} & 0 \end{array} & \begin{array}{cc} \ldots & \frac{l_{g.h}}{l_{gI_{g}.}} \end{array}\\ \begin{array}{cc} \frac{l_{g.h^{\prime }}}{l_{g1}.} & 0 \end{array} & \begin{array}{cc} \ldots & \frac{l_{g.h^{\prime }}}{l_{gI_{g}.}} \end{array} \end{array}\right]$$

It is obvious that ‘always showing together’ means that *l*_*gih*_/*l*_*gi*._ and *l*_*gih’*_/*l*_*gi*_., *i* = 1,…, *I*_*g*_, are either both zeros or non-zero numbers with values *l*_*g*.*h*_/*l*_*gi*._ and *l*_*g*.*h’*_/*l*_*gi*._. So the ‘always showing together’ read types will have their corresponding rows in *P*_*g*_ proportional to each other. Then we could just conduct one hypothesis testing by combining these ‘always showing together’ read types.

**Fig 2:**
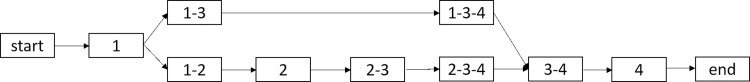
An example splicing graph for model simplification. The ‘start’ and ‘end’ nodes are pseudo nodes. In this example, there are four exons represented by 1, 2, 3, and 4. Each node represents a possible read type, and nodes are connected in a way that a node shares the same starting exons with a node to its left and shares the same ending exons with a node to its right. An arrow connecting two nodes points to the end direction. A4 in S1 File delineates the steps of how to construct a splicing graph.

To find the read types that are ‘always showing together’, we could use a splicing graph model. The line graph model [14, 15] is appropriate for this goal. Moreover, pseudo start and end nodes provide a possibility to modify the complexity of the graph by adding or removing potential paths between other true nodes and the pseudo start/end nodes. In our method, a simple graph model was used so that the start node connects to only the left most node and only the right most node connects to the end node (Fig 2).

If the splicing graph model correctly reflects the connection between nodes, we can get the following result: If two nodes are connected and the out degree of the smaller node (in lexicographic order) equals the in degree of the larger node, which is 1, then these two types of read must ‘always showing together’. The reason is that, given two types of read *h* < *h*’ satisfying this condition, if there is an isoform can generate *h* but not *h*’, there must be another path coming out from *h* to a node other than *h*’; if there is an isoform can generate *h*’ but not *h*, there must be another path coming into *h*’ from a node other than *h*. Strategies to construct a line graph model based on existing read types are given in A4 in S1 File.

## 3. Results

### 3.1 Simulation setting

Extensive simulations were performed to compare our proposed method SGNB with popularly used edgeR and DESeq without pre-specifying any isoform structures. Without loss of generality, we simulated single-end RNA-seq reads from an R package–‘polyester’ [16] using about 1800 genes from human chromosome 1. This R package ‘polyester’ simulates RNA-seq count data mimicking all sequencing steps from cutting transcripts into short fragments, then sequencing one end of the fragments to get raw RNA-seq data.

We assume the distribution of isoforms’ copy numbers to negative binomial, and used a large variance to cover a large range of copy numbers. A negative binomial distribution with *μ* = 10 and *σ*^2^ = 210 was chosen to randomly create the isoforms’ copy numbers. The average fragment length in ‘polyester’ was set to 250 base pairs with a standard error of 25 base pairs. The read length was set to be 100 base pairs. Since it is estimated that there were 20,000 protein coding genes and a total 20 × 10^6^ of RNA-seq reads is usually normal, we set 1000 RNA-seq reads for each gene. 1.8 × 10^6^ total RNA-seq reads were generated for 1800 genes. We assigned the same number of subjects to each condition (e.g. normal and disease), and let it range from 5 to 20 with a step size of 5 (i.e. sample size N = 10, 20, 30 and 40). The default setting of dispersion parameter in ‘polyester’ for controlling per-transcript mean/variance relationship in ‘polyester’ is ‘3 / reads per transcript’, which generates a low-variance situation. The dispersion in reality may vary in a large range, therefore we simulated data using two dispersion parameters, a small one (‘5 / reads per transcript’) and a large one (‘30 / reads per transcript’).

For each simulation scenario, 10 datasets were generated to evaluate the model performance. Type I errors were assessed by setting all genes to be non-differentially expressed. For power comparison, ~30% of genes were set to be the DE genes, which means that for each of these genes, there was at least one of its isoforms having different copies under different conditions. For the isoforms of DE genes, we sampled their log fold changes from a normal distribution with *μ* = 0 and *σ*^2^ = 1 which implies relatively small fold changes. Our intention is to better differentiate methods in this case as all methods are very sensitive to a large fold change which will give us a perfect power curve.

For computational efficiency, a smaller-scale simulation study was performed to confirm if our method is more sensitive in detecting expression level change at isoform-level than edgeR and DESeq. In this simulation, the ‘whole transcriptome’ contains 10 genes and 1 of them (has 2 isoforms with similar length) is the DE gene. All rest simulation parameters are the same with other larger-scale simulations, except the copy numbers of isoforms for the DE gene. In order to change the isoform-wise expression while keeping the total size of isoforms similar across two conditions, we set the copy numbers of the DE gene’s isoforms to be 10 and 50 under one condition and 50 and 10 under another condition.

### 3.2 Simulation results

**Fig 3:**
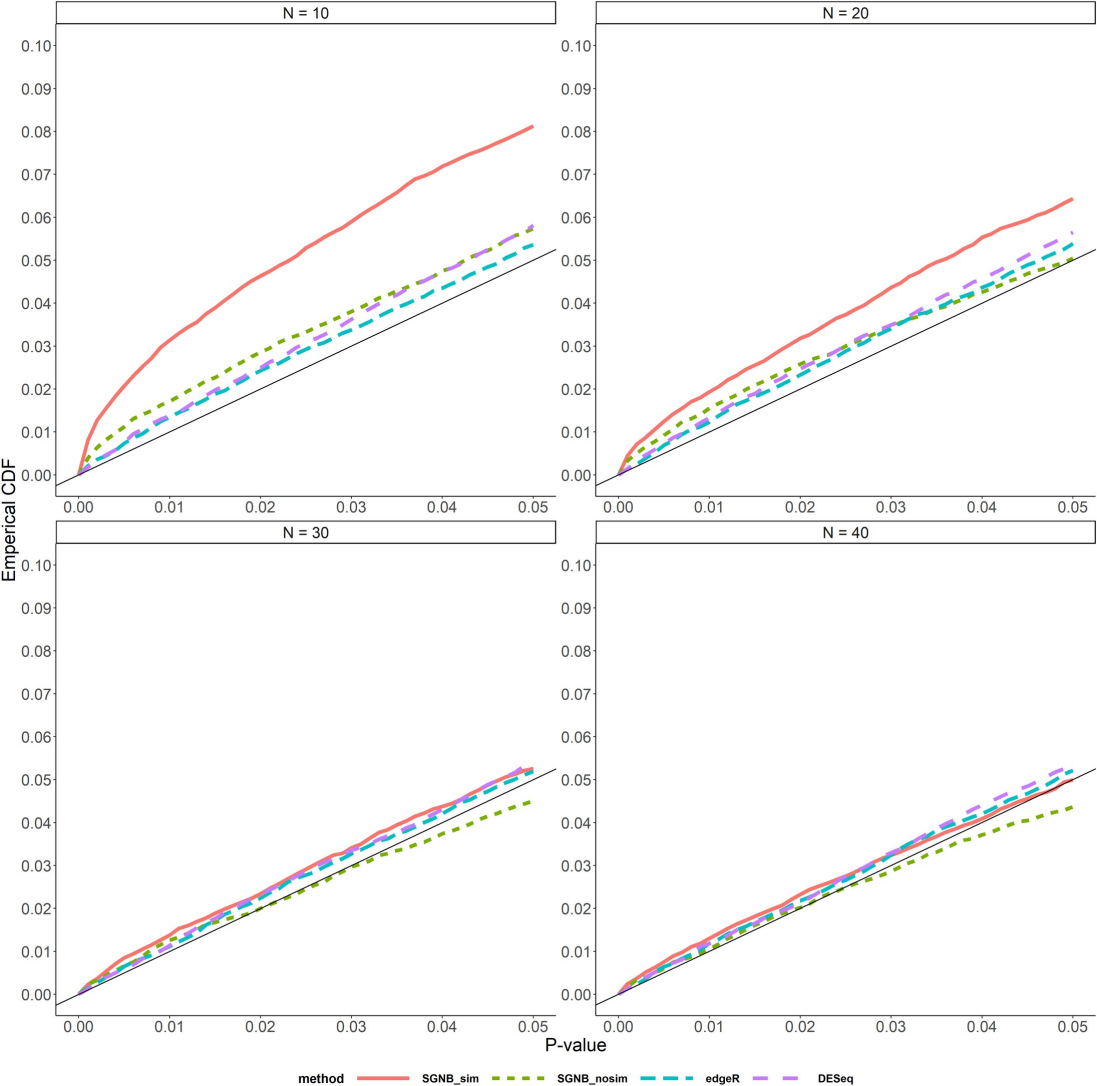
Type I error control with small dispersion by P-value < 0.05.

**Fig 4:**
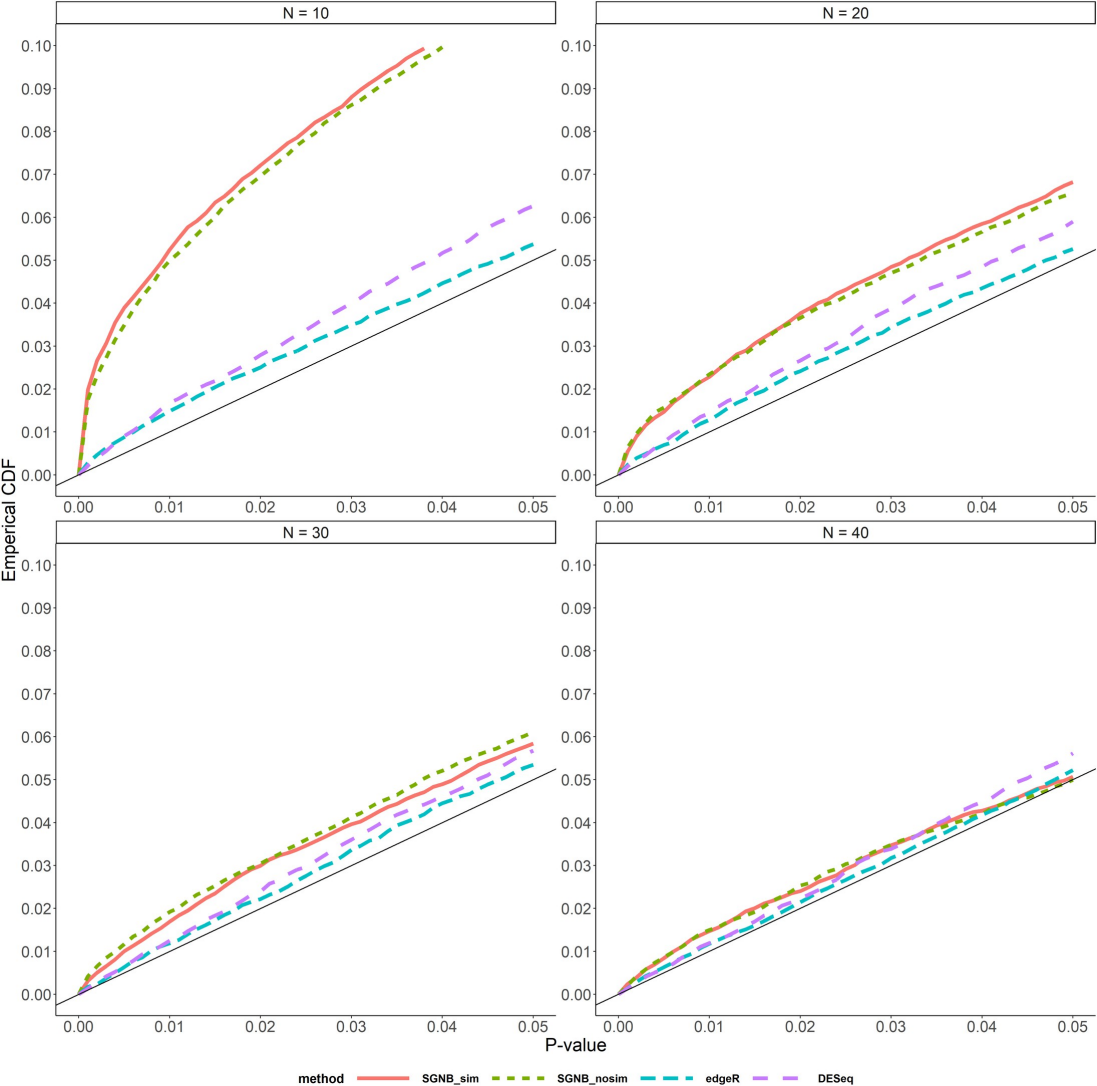
Type I error control with large dispersion by P-value < 0.05.

Firstly, we evaluated the performances of type I error control across different models (SGNB with model simplification, SGNB without model simplification, edgeR and DESeq) by comparing the true false positive rate with different p-value thresholds between 0 and 0.05 (Fig 3 for small dispersion and Fig 4 for large dispersion).

In Figs 3 and 4, the red solid line is for SGNB with model simplification and the green dash line for SGNB without model simplification. Figs 3 and 4 suggest that when the dispersion is small, SGNB without model simplification performs better than SGNB with model simplification; and when the dispersion is large, they have the similar performance. Compared with edgeR and DESeq, SGNB with model simplification only can achieve a similar type I error control performance when the sample size is large, while edgeR and DESeq have a good type I error control across all the sample size settings. Also, SGNB with model simplification needs more samples to control type I error under the large dispersion setting than the small dispersion setting. This suggests that SGNB is more suitable for studies with sample size > 30.

**Fig 5:**
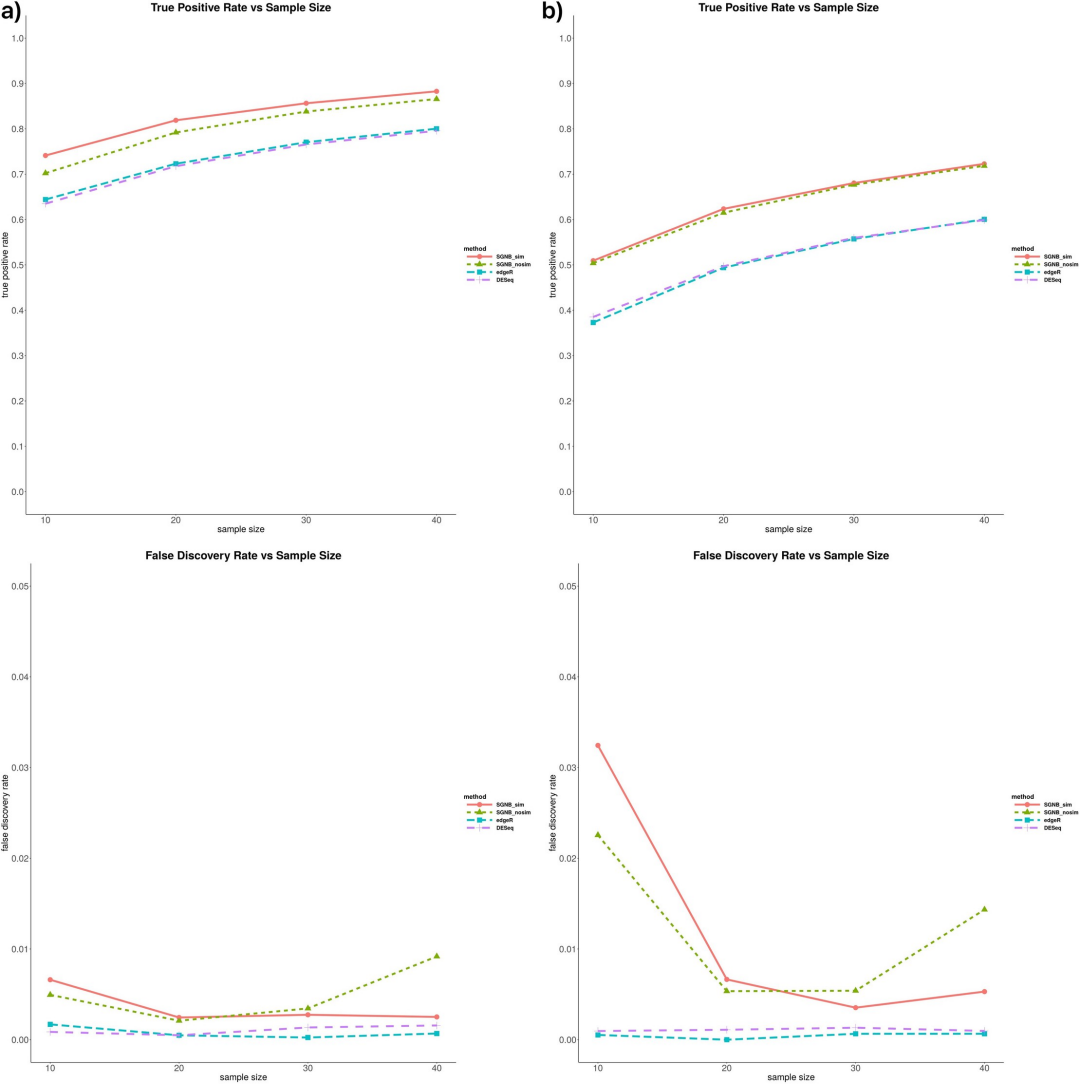
False discovery rate and true positive rate vs. sample size at 0.05 significance level. a) Small dispersion. b) Large dispersion.

Secondly, we compared average false discovery rate (FDR) and true positive rate (TPR) among these 4 methods when DE genes exist from the 10 simulation runs at different sample-size setting (Fig 5, exact numerical values can be found in A3 in S1 File). We selected DE genes at a significance level of 0.05 with Bonferroni adjustment for the number of genes. These figures suggested that both SGNBs (with and without model simplification) had a higher TPR than edgeR and DESeq at the expense of a slightly higher FDR. TPR was increased about 10 percent by SGNB than edgeR and DESeq while there was only a tiny increase in FDR. SGNB with model simplification always has a higher TPR than SGNB without the model simplification.

**Fig 6:**
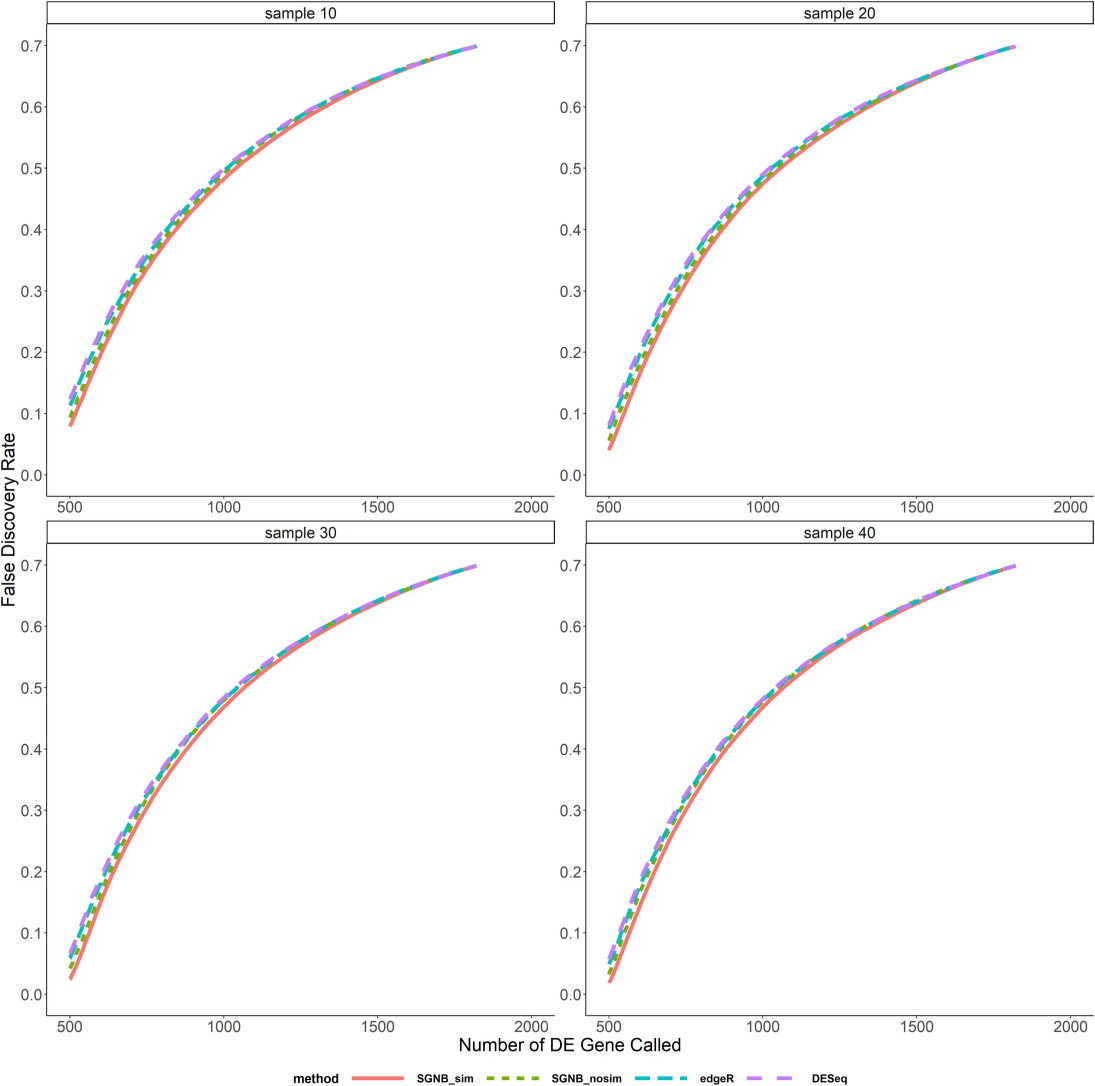
False discovery rate vs. number of DE gene called with small dispersion.

**Fig 7:**
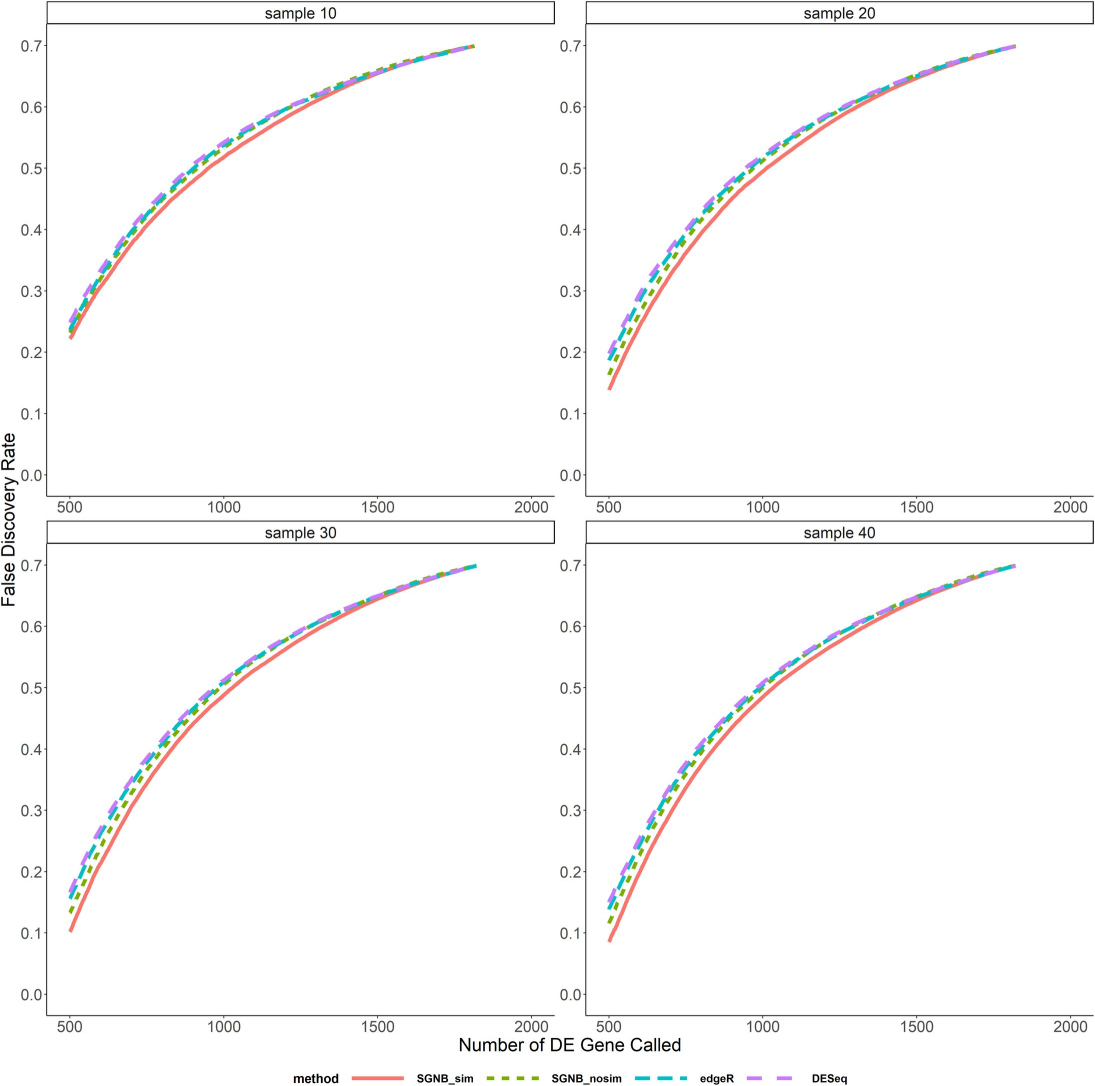
False discovery rate vs. number of DE gene called with large dispersion.

**Fig 8:**
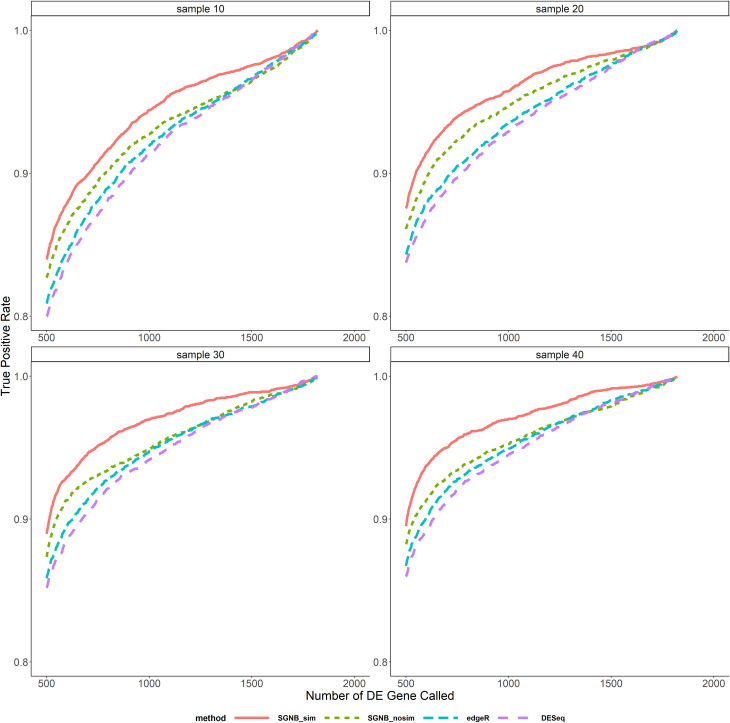
True positive rate vs. number of DE gene called with small dispersion.

**Fig 9:**
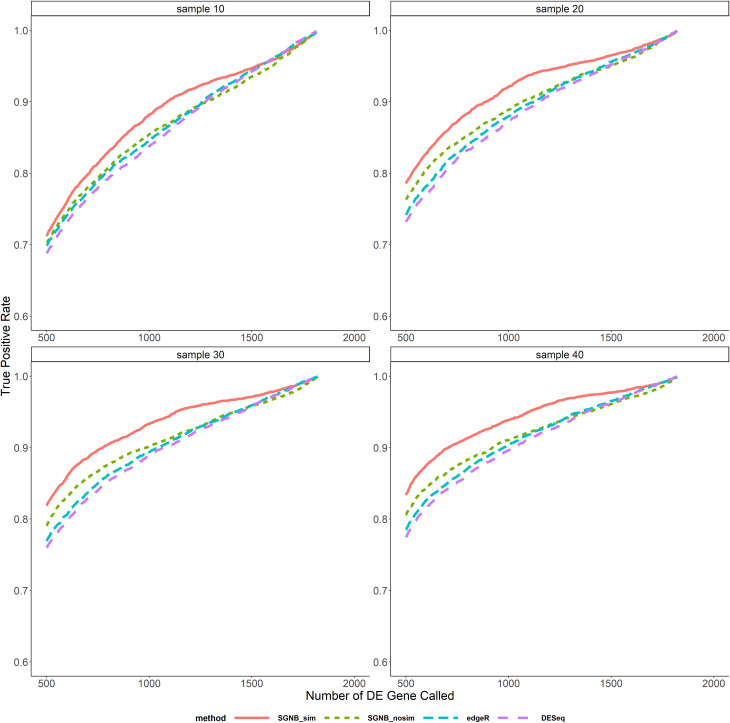
True positive rate vs. number of DE gene called with large dispersion.

In the comparison above, different methods called different number of DE genes at the same pre-specified significant level. We also compared the average FDR and TPR against the same number of DE genes called as shown in Figs 6–9. It clearly shows that given the same fixed number of DE genes called, SGNB outperforms edgeR and DESeq in terms of having lower FDR and higher TPR.

Finally, we had a simulation setting to compare the capabilities of SGNB, edgeR and DESeq in detecting isoform-wise expression level change. Among 10 genes, there was 1 DE gene whose total size of isoforms kept unchanged but the isoform-level expression changed. This gene has 2 isoforms. The SGNB with model simplification detected it 100 times during 100 runs, while the edgeR and DESeq detected it only once during 100 runs.

### 3.3 Real data analysis

The real data comes from a published study as in [17]. There are 17 samples from human classical monocyte subsets and 17 samples from nonclassical monocyte subsets. The author compared the DE genes called by different methods combinations against a reference DE gene dataset which comes from the DE analysis of 4 published microarray datasets. However, these DE genes may not be a good reference for our analysis, since they are detected under a gene-level analysis without considering isoforms.

**Fig 10:**
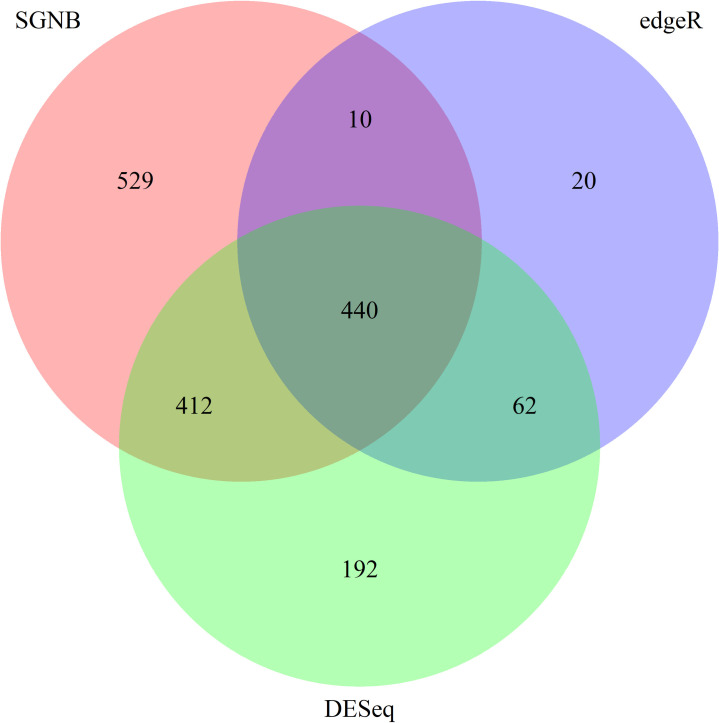
Venn diagram of DE genes detected using different methods. Bonferroni adjustment was made at the significance level of 0.05.

**Fig 11:**
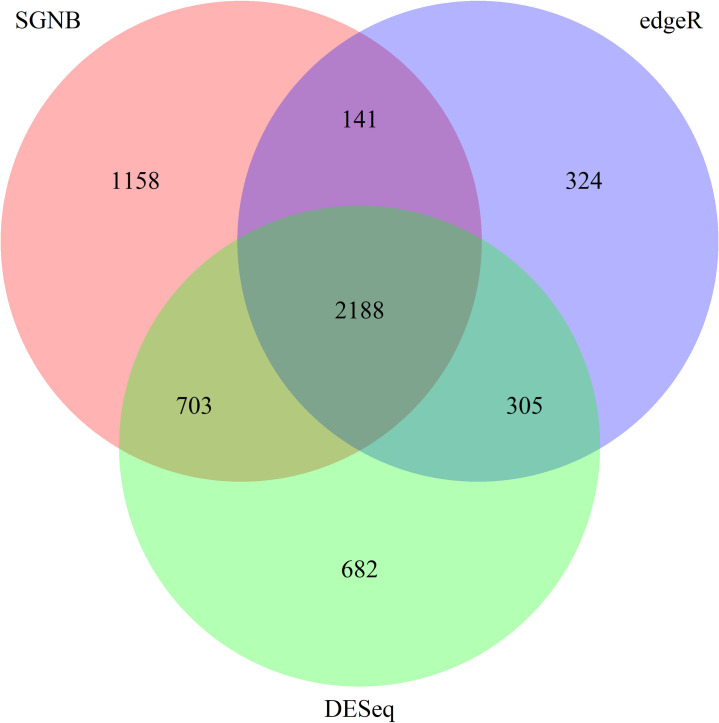
Venn diagram of DE genes detected using different methods and controlling FDR at 5% [18].

We mapped the RNA-seq data with TopHat. DE genes were called at 0.05 and 0.01 significance levels with Bonferroni adjustment. A Venn diagram is generated to compare DE genes called by edgeR, DESeq, and SGNB with model simplification (Fig 10 using significance level of 0.05 and Fig 11 from controlling FDR at 5%). SGNB without model simplification was not used since it was not as good as SGNB with model simplification as suggested by our simulation studies.

At the significance level of 0.05, as shown in Fig 10, there are 1391 genes called as DE genes by SGNB, 532 genes called by edgeR, and 1106 genes called by DESeq. The number of common genes called by SGNB and edgeR is 450, which is 84.6% of genes called by edgeR. The number of common genes called by SGNB and DESeq is 852, which is 77.0% of genes called by DESeq. Therefore, SGNB also detected majority of genes called by the other two methods in addition to detecting 529 genes that were not called by the other two methods. Similar findings can be found from Fig 11 using a significance level of 0.01 and Bonferroni adjustment.

## 4. Discussion

In this paper, we proposed a new isoform-free model, SGNB, for detecting DE genes at the isoform-level. This method is more sensitive to the level change of isoform-wise expression, while keeping the benefit of a gene-level method that does not require pre-specifying the isoform structure. Although our method needs to use the gene annotation file, we only need to know exons’ ranges but not the exact isoforms’ structures. Based on gene annotation file, we can perform test on the unique read level and use Bonferroni adjustment to get a gene-level p-value in order to maintain the overall type-I error control at the gene-level. We compared our method with edgeR and DESeq in terms of type I error control, true positive rate, and false discovery rate. At the expense of slightly increasing false discovery rate, our new method can dramatically increase true positive rate due to its capability in identifying isoform-wise change in expression level.

From the enlarged type I error control graphs (Figs 3 and 4), we see that our proposed method SGNB with model simplification had slightly inflated type I error compared to edgeR and DESeq when sample size is small (e.g. N<30). But when the sample size is large (e.g. N≥30), SGNB with model simplification performs similarly as edgeR and DESeq. So we suggest that our method should be used with a large sample size. Right now with quick advance in technology, it is neither too expensive nor time consuming to get RNA-seq data and hence a large sample size in practice becomes more and more feasible.

The model simplification procedure is an important feature of our method. It can not only save the computing time by reducing a big number of parameters to be estimated and hypothesis tests to be tested (reduced the number of hypothesis from about 47,000 to 17,000 on average based on our simulations), but also have benefits on improving FDR and TPR. From Fig 5A and 5B, we can see that SGNB with model simplification always had a little bit higher TPR than SGNB without model simplification and a lower FDR when the sample size is large (N≥30). The similar benefits can be seen from Figs 6–9. With the same number of DE genes called, SGNB with model simplification consistently had a higher TPR and a lower FDR.

The key idea in model simplification is to combine the rows in matrix *P*_*g*_ according to a splicing graph while keeping its column rank as full. We show that when the original *P*_*g*_ is a full rank matrix, the hypothesis (3) and (4) will be equivalent. It’s hard to prove that under what condition *P*_*g*_ would be a full rank matrix, but it should be a reasonable assumption since that matrix usually has a large number of rows (equaling to the number of read types) and a small number of columns (equaling to the number of isoforms). Moreover, the splicing graph built based on our algorithm may not be exactly accurate because of the complicated unknown structure of isoforms and we may even wrongly combine read types whose rows are not proportional to each other in matrix *P*_*g*_. However, as long as we can keep the rank of matrix *P*_*g*_, wrongly combined read types should not affect the hypothesis testing. Our simulations have shown that the model simplification procedure works well, since there is no power reduction compared to the model without simplification but saving much time in computation.

Currently there are other methods proposed to improve on the disadvantage of using only the total sizes of the isoforms of a gene for calling a DE gene (e.g., DEXseq from [19]; diffSpliceDGE from [20]). DEXseq tests for differential exon usage as defined as number of transcripts from the gene that contain this exon/number of all transcripts from the gene. It can then call a DE gene if it contains at least one differentially used exon. Similarly, diffSpliceDGE also detects differential exon usage using a negative binomial generalized log-linear model fit at the exon level. Our method will be another one added to this pool of methods that researchers may use. We applied all three methods to our real data example for a comparison. Our method detected a different set of DE genes from DEXseq and diffSpliceDGE as expected. But both at the significance level of 1% with Bonferroni adjustment and controlling FDR at 1%, our method actually reported more number of DE genes. The detailed information is listed in A5 in S1 File. However, validity of the additional DE genes detected by our method needs to be confirmed by biological experiments. In addition, further evaluation and comparison of our method with other methods that use known isoform structures are very interesting and we plan to pursue hem in more detailed and comprehensive future analyses. We have put our proposed method in an R package ‘SGNB’ and the example R scripts used for real data analysis can be found in A6 in S1 File. We also used such R scripts for detailed explanation of how to use main function fit_SGNB_exact() in the readme and vignette file of our R package.

Our method is best suited for the single-end RNA-seq reads. For paired-end reads, since we do not know what nucleotides are there in the insertion part, it will be hard to summarize a paired-end read to a read type. However, we can simply treat it as two single-end reads by ignoring the insertion information of the paired-end reads, which may lead to a non-efficient use of the data. Research is ongoing to have a model that is also well suited for paired-end reads.

## Supporting information

S1 File (DOCX)

S2 File (TXT)
