# Supplementary material for: A model for isoform-level differential expression analysis using RNA-seq data without pre-specifying isoform structure
Source: PLoS One. 2022 May 16;17(5):e0266162. doi: 10.1371/journal.pone.0266162 (PMC9109925; doi:10.1371/journal.pone.0266162)
Supplement: S1 File — (DOCX) [file pone.0266162.s001.docx]

**A1. Parameter Estimation**

Given

$$X_{eghj}\sim NB(N^{*}\theta_{egh},\varphi_{gh})$$

$$Z_{egh}=\sum_{j=1}^{J_{e}} X_{eghj}\sim NB(J_{e}N^{*}\theta_{egh},\varphi_{gh}J_{e}^{-1})$$

The conditional likelihood function for $\varphi_{gh}$ is

| $\sum_{e=0}^{1} \left[ \sum_{j=1}^{J_{e}} log\Gamma\left( x_{eghj}+\varphi_{gh}^{-1} \right)+log\Gamma\left( J_{e}\varphi_{gh}^{-1} \right)-log\Gamma\left( z_{egh}+J_{e}\varphi_{gh}^{-1} \right)-J_{e}log\Gamma\left( \varphi_{gh}^{-1} \right) \right]$ | (1) |
| --- | --- |

| Algorithm for estimating dispersion parameters $\varphi_{gh}$ | |
| --- | --- |
| Step 1. | Calculate $N^{*}=\left( \prod_{j=1}^{J_{0}} N_{j}^{*}\prod_{j=1}^{J_{1}} N_{j}^{*} \right)^{\frac{1}{J_{0+J_{1}}}}$ |
| Step 2. | Initialize $\varphi_{gh}$ by assuming $X_{eghj}$ have the same library size $N^{*}$ and maximizing (1) |
| Step 3. | Given current $\hat{\varphi}_{gh}$, estimate $\theta_{egh}$ by EM algorithm. |
|  | E step:  $Q\left( \theta_{egh}\vert\theta_{egh}^{(t)} \right)=\sum_{e=0}^{1} \sum_{j=1}^{J_{e}} \left[ -\hat{\varphi}_{gh}^{-1}log\left( \theta_{egh} \right)-\left( \theta_{egh}\hat{\varphi}_{gh} \right)^{-1}\left( x_{eghj}+\hat{\varphi}_{gh}^{-1} \right)\left( \frac{\theta_{egh}^{(t)}\hat{\varphi}_{gh}}{N_{j}^{*}\theta_{egh}^{(t)}\hat{\varphi}_{gh}+1} \right) \right]$ |
|  | M step:  $\hat{\theta}_{egh}=\frac{\sum_{j=1}^{J_{e}} \left[ \left( x_{eghj}+\hat{\varphi}_{gh}^{-1} \right)\left( \frac{\theta_{egh}^{(t)}\hat{\varphi}_{gh}}{N_{j}^{*}\theta_{egh}^{(t)}\hat{\varphi}_{gh}+1} \right) \right]}{J_{e}}$ |
| Step 4. | Assume $X_{eghj}\sim NB\left( N_{j}^{*}\hat{\theta}_{egh},\hat{\varphi}_{gh} \right)$, calculate $p_{j}=P\left( X_{eghj}<x_{eghj};N_{j}^{*}\hat{\theta}_{egh},\hat{\varphi}_{gh} \right)+\frac{1}{2}P\left( X_{eghj}=x_{eghj};N_{j}^{*}\hat{\theta}_{egh},\hat{\varphi}_{gh} \right)$ |
| Step 5. | Generate $X_{eghj}^{pseudo}\sim NB(N^{*}\hat{\theta}_{egh},\hat{\varphi}_{gh})$ with $p_{j}=P\left( X_{eghj}^{pseudo}\leq x_{eghj}^{pseudo} \right)$ |
| Step 6. | Update $\varphi_{gh}$ by (1) with pseudo data $X_{eghj}^{pseudo}$ |
| Step 7. | Repeat Step 3 – Step 6 until $\varphi_{gh}$ converges. |

**A2. P-value Calculation for One Read Type.**

$$Pvalue=2\times min\left( P\left( Z_{0gh}\leq z_{0gh}|z_{gh} \right),P\left( Z_{0gh}\geq z_{0gh}|z_{gh} \right) \right)$$

$$P\left( Z_{0gh}=z_{0gh}|z_{gh} \right)=\frac{\Gamma\left( z_{0gh}+J_{0}\hat{\varphi}_{gh}^{-1} \right)\Gamma\left( z_{gh}-z_{0gh}+J_{1}\hat{\varphi}_{gh}^{-1} \right)\Gamma\left( \left( J_{0}+J_{1} \right)\hat{\varphi}_{gh}^{-1} \right)\Gamma\left( z_{gh}+1 \right)}{\Gamma\left( J_{0}\hat{\varphi}_{gh}^{-1} \right)\Gamma\left( z_{0gh}+1 \right)\Gamma\left( J_{1}\hat{\varphi}_{gh}^{-1} \right)\Gamma\left( z_{gh}-z_{0gh}+1 \right)\Gamma\left( z_{gh}+\left( J_{0}+J_{1} \right)\hat{\varphi}_{gh}^{-1} \right)}$$

**A3. Empirical Type I Error at 0.05 Significant Level, TPR and FDR Table Corresponding to Figures 4, 5 and 6.**

Table: Mean and Standard Deviation of TPR and FDR for 10 simulation Runs at 0.05 Significance Level after Bonferroni Adjustment.

| Dispersion | Sample Size | Method | Type I Error | | TPR | | FDR | |
| --- | --- | --- | --- | --- | --- | --- | --- | --- |
|  |  |  | Mean | Sd | Mean | Sd | Mean | Sd |
| Small Dispersion | 10 | SGNB with model simplification | 0.081 | 0.008 | 0.741 | 0.021 | 0.0021 | 0.0011 |
|  |  | SGNB without model simplification | 0.057 | 0.008 | 0.702 | 0.026 | 0.0015 | 0.0011 |
|  |  | edgeR | 0.054 | 0.006 | 0.644 | 0.024 | 0.0005 | 0.0004 |
|  |  | DESeq | 0.058 | 0.009 | 0.635 | 0.025 | 0.0002 | 0.0004 |
|  | 20 | SGNB with model simplification | 0.064 | 0.008 | 0.819 | 0.019 | 0.0009 | 0.0009 |
|  |  | SGNB without model simplification | 0.050 | 0.012 | 0.792 | 0.018 | 0.0007 | 0.0008 |
|  |  | edgeR | 0.054 | 0.005 | 0.723 | 0.020 | 0.0002 | 0.0003 |
|  |  | DESeq | 0.056 | 0.005 | 0.718 | 0.021 | 0.0002 | 0.0003 |
|  | 30 | SGNB with model simplification | 0.053 | 0.005 | 0.856 | 0.016 | 0.0010 | 0.0006 |
|  |  | SGNB without model simplification | 0.045 | 0.004 | 0.838 | 0.013 | 0.0013 | 0.0012 |
|  |  | edgeR | 0.052 | 0.006 | 0.771 | 0.015 | 0.0001 | 0.0002 |
|  |  | DESeq | 0.054 | 0.005 | 0.766 | 0.017 | 0.0005 | 0.0008 |
|  | 40 | SGNB with model simplification | 0.050 | 0.008 | 0.883 | 0.014 | 0.0009 | 0.0009 |
|  |  | SGNB without model simplification | 0.044 | 0.003 | 0.866 | 0.012 | 0.0034 | 0.0017 |
|  |  | edgeR | 0.052 | 0.005 | 0.800 | 0.016 | 0.0002 | 0.0004 |
|  |  | DESeq | 0.053 | 0.005 | 0.796 | 0.017 | 0.0006 | 0.0008 |
| Large Dispersion | 10 | SGNB with model simplification | 0.113 | 0.007 | 0.509 | 0.022 | 0.0073 | 0.0029 |
|  |  | SGNB without model simplification | 0.112 | 0.008 | 0.503 | 0.023 | 0.0050 | 0.0024 |
|  |  | edgeR | 0.054 | 0.005 | 0.373 | 0.021 | 0.0001 | 0.0002 |
|  |  | DESeq | 0.063 | 0.007 | 0.385 | 0.024 | 0.0002 | 0.0003 |
|  | 20 | SGNB with model simplification | 0.068 | 0.008 | 0.624 | 0.022 | 0.0018 | 0.0014 |
|  |  | SGNB without model simplification | 0.066 | 0.005 | 0.615 | 0.027 | 0.0014 | 0.0010 |
|  |  | edgeR | 0.053 | 0.004 | 0.494 | 0.022 | 0 | NA |
|  |  | DESeq | 0.059 | 0.005 | 0.497 | 0.019 | 0.0002 | 0.0004 |
|  | 30 | SGNB with model simplification | 0.058 | 0.008 | 0.681 | 0.020 | 0.0010 | 0.0006 |
|  |  | SGNB without model simplification | 0.061 | 0.009 | 0.677 | 0.021 | 0.0016 | 0.0009 |
|  |  | edgeR | 0.053 | 0.004 | 0.557 | 0.022 | 0.0002 | 0.0003 |
|  |  | DESeq | 0.057 | 0.005 | 0.560 | 0.022 | 0.0003 | 0.0004 |
|  | 40 | SGNB with model simplification | 0.051 | 0.004 | 0.723 | 0.014 | 0.0016 | 0.0013 |
|  |  | SGNB without model simplification | 0.050 | 0.006 | 0.719 | 0.018 | 0.0045 | 0.0017 |
|  |  | edgeR | 0.052 | 0.004 | 0.600 | 0.022 | 0.0002 | 0.0003 |
|  |  | DESeq | 0.056 | 0.002 | 0.599 | 0.022 | 0.0002 | 0.0004 |

**A4. Strategies to Construct A Splicing Graph.**

For a given gene $g$, we denote the set of exon IDs as $E=\left\{ i|i=1,2,\ldots,E_{g} \right\}$, where $E_{g}$ is the total number of unique exons of gene $g$. The order of the IDs shows the position relationship of exons, that is, if $i_{1}<i_{2}, \forall i_{1},i_{2}\in E$, then the starting position of exon $i_{1}$ is before the starting position of exon $i_{2}$. Suppose we have two read types $r$ and $r^{'}$, which can be represented by a string of exon IDs, $i_{1}i_{2},\ldots i_{n}$ and $i_{1}^{'}i_{2}^{'}\ldots i_{n}^{'}$ respectively.

*Definition 1. Prefix-Substring: any substring start from the left most position of a read type string, e.g.* $i_{1}i_{2}\ldots i_{k},\forall1\leq k\leq n$*.*

*Definition 2. Suffix-Substring: any substring end at the right most position of a read type string, e.g.* $i_{k},i_{k+1},\ldots i_{n},\forall1\leq k\leq n$*.*

Let’s denote the set of all prefix-substring of read type $r$ as $PS_{r}$ and denote the set of all suffix-substring as $SS_{r}$.

We say that $r$ can connect to $r^{'}$ in a splicing graph if $r<r^{'}$(in dictionary order) and $SS_{r}\cap PS_{r^{'}}\neq\emptyset$, and denote it as $r\to r^{'}$. Moreover, given $r\to r^{'}$ and $r\to r^{''}$, if $r^{'}<r^{''}$ and $r^{'}\to r^{''}$, then we will not connect $r$ to $r^{''}$.

**A5. The results from using DEXseq and diffSpliceDGE on the real data example**

26343 genes with multiple exons were used while applying DEXseq and diffSpliceDGE as they only work with genes with multiple exons. At 1% FDR and 1% type I error after Bonferroni adjustment, Figure A5 shows that three methods detected different sets of DE genes as expected and our proposed SGNB methods had slightly more number of DE genes detected.

(a) (b)


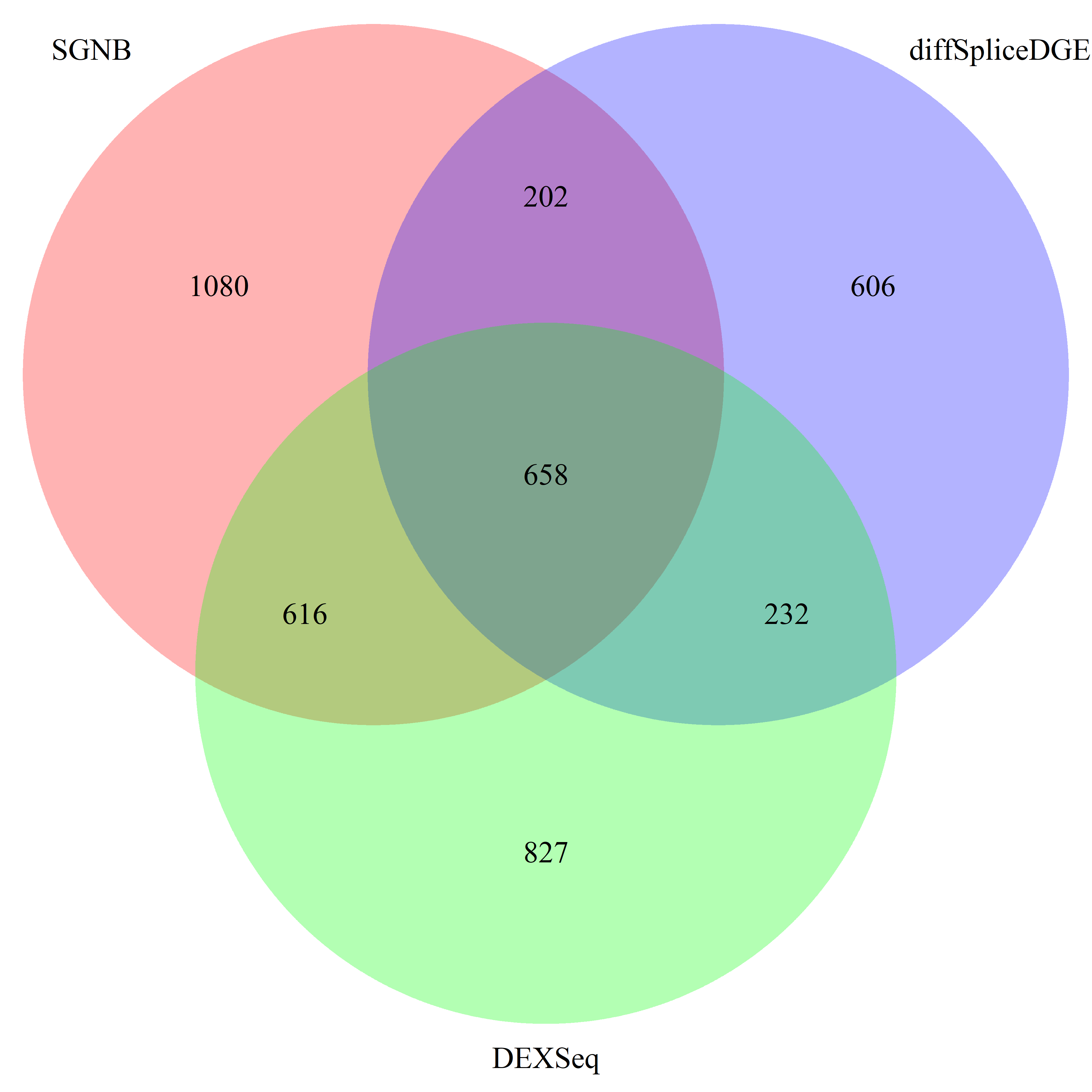

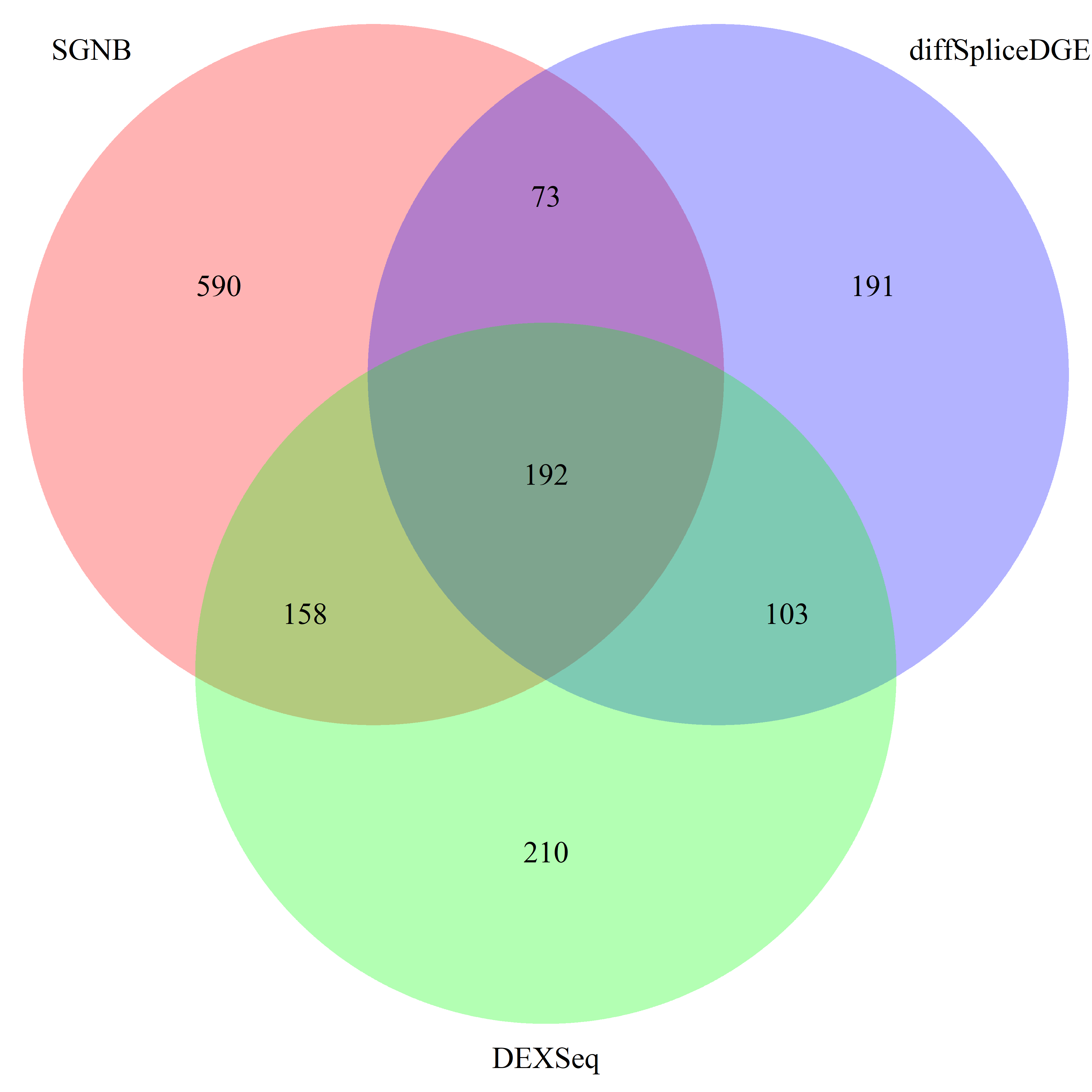


Figure A5: Venn Diagram of DE Genes detected using our proposed method SGNB, DEXseq and iffSpliceDGE with (a) controlling FDR at 1% and (b) controlling type I error at 1% with Bonferroni adjustment.

**A6. R codes used for analyzing real data example**

#!/usr/bin/Rscript

rm(list = ls())

require(edgeR)

require(DESeq2)

require(SGNB)

require(data.table)

require(Rsubread)

# summarize mapped reads into count table------------------------------------------------------

#sum_read_SGNB <- summarize_read_single_end("./group0/", "./group1/")

load('./sum_read_SGNB.RData')

file_group0 <- list.files("./group0/", pattern = "\\.sam", full.names = TRUE)

file_group1 <- list.files("./group1/", pattern = "\\.sam", full.names = TRUE)

files <- c(file_group0, file_group1)

sum_read_rev <- featureCounts(files, annot.ext = './Gene_Annotation/Homo_sapiens.GRCh38.80.gtf',

isGTFAnnotationFile = TRUE, minOverlap = 50, allowMultiOverlap = TRUE)

sum_read_edgeR <- as.data.frame(sum_read_rev$counts)

rs <- rowSums(sum_read_edgeR)

sum_read_edgeR <- sum_read_edgeR[which(rs != 0), ]

# analyze------------------------------------------------------------------------

# SGNB

res_SGNB <- fit_SGNB_exact(sum_read_SGNB)

res_SGNB$p_value_ad <- p.adjust(res_SGNB$p_value, method = 'BH')

# edgeR

group0_samp_num <- length(file_group0)

group1_samp_num <- length(file_group1)

edgeR_group <- factor(c(rep(1, group0_samp_num), rep(2, group1_samp_num)))

edgeR_y <- DGEList(counts = sum_read_edgeR, group = edgeR_group)

edgeR_y <- calcNormFactors(edgeR_y)

edgeR_design <- model.matrix(~ edgeR_group)

edgeR_y <- estimateDisp(edgeR_y, edgeR_design)

edgeR_et <- exactTest(edgeR_y)

res_edgeR <- data.frame(gene = rownames(edgeR_et$table), edgeR_et = edgeR_et$table[[3]])

res_edgeR$edgeR_et_ad <- p.adjust(res_edgeR$edgeR_et, method = 'BH')

# DESeq2

colData <- data.frame(condition = factor(c(rep(1, group0_samp_num), rep(2, group1_samp_num))))

DESeq_y <- DESeqDataSetFromMatrix(countData = sum_read_edgeR, colData = colData, design = ~ condition)

DESeq_fit <- DESeq(DESeq_y)

res_DESeq_pre <- results(DESeq_fit)

res_DESeq <- data.frame(gene = rownames(res_DESeq_pre), DESeq_p = res_DESeq_pre$pvalue)

res_DESeq$DESeq_p_ad <- p.adjust(res_DESeq$DESeq_p, method = 'BH')

# summarize results--------------------------------------------------------------------------

results <- merge(res_SGNB, res_edgeR, by.x = 'gene_id', by.y = 'gene', all = TRUE)

results <- merge(results, res_DESeq, by.x = 'gene_id', by.y = 'gene', all = TRUE)

save(results, file = './comp_real_results.RData')
